# The Effect of Endotracheal Tube Cuff Shape on Post-extubation Sore Throat in Critically Ill Patients in a Rural Tertiary Care Hospital

**DOI:** 10.7759/cureus.42519

**Published:** 2023-07-26

**Authors:** Akshaya Venkitesh, Anson Angel Nelson, Akshaya N Shetti

**Affiliations:** 1 Department of Anatomy, Dr. Balasaheb Vikhe Patil Rural Medical College, Pravara Institute of Medical Sciences (PIMS), Loni, IND; 2 Department of Anaesthesiology, Dr. Balasaheb Vikhe Patil Rural Medical College, Pravara Institute of Medical Sciences (PIMS), Loni, IND

**Keywords:** sore throat, tapered shape, extubation, endotracheal tube, cuff

## Abstract

Background: Patients admitted to the critical care unit often require extended periods of mechanical ventilation. After extubation, patients often report discomfort in their throats, coughing, and hoarseness of voice. These symptoms have been linked to the shape of the cuff on the endotracheal tube and are described in terms of the surface area of the cuff in contact with the trachea.

Methods: During this pilot study, 160 adults receiving intensive primary care were randomly assigned to one of two groups (Gathering A or Gathering B; 80 patients each). Intubated patients were separated into two groups: Group C consisted of those who wore a looser, barrel-shaped sleeve, and Group T consisted of those who wore a more restrictive sleeve. The severity of post-extubation side effects was assessed, including sore throat, dry voice, and hack, and the occurrence of these symptoms was also documented.

Result: Neither the number of intubation attempts nor the experience level of the residents who performed them differed significantly between the two groups (p > 0.05). A smaller percentage of patients in Gathering T experienced sore throats in the first, 12^th^, and 24^th^ hours after extubation compared to patients in Group C at these same time points (p = 0.05). With time passing, fewer people in Group C and Group T experienced hoarseness of voice after extubation. There is a declining trend in the incidence of cough post-extubation in Group T, as compared to an initial increase in the trend for cough post-extubation with a gradual decline as time progressed in Group C.

Conclusion: There is an overall decrease in the incidence of post-extubation emergence phenomena with tapered shape cuffed endotracheal tubes when compared with conventional cylindrical type cuffed endotracheal tubes.

## Introduction

Intubation is a standard procedure in both the operating room (OR) and the critical care unit (CCU). An unpleasant side effect of endotracheal intubation is a painful throat after extubation observed at a rate of 21% and as high as 74% [1,2]. Intensive care unit (ICU) patients typically remain intubated for longer periods of time than those in the OR, making it all the more crucial to keep endotracheal cuff pressure within acceptable ranges. The cuff of an endotracheal tube (ETT) serves two purposes: sealing off the airway to avoid aspiration and maintaining positive pressure breathing [3]. There is undeniable evidence that subglottic discharges containing germs enter the trachea via the cylinder sleeve of a ventilator, causing ventilator-associated pneumonia (VAP). Patients undergoing mechanical ventilation benefit greatly from having an enlarged endotracheal tube sleeve in place since this reduces the risk of gas leakage and the aspiration of oropharyngeal contents into the lungs [4]. A sore throat can be brought on by a number of factors, including failed endotracheal intubation attempts, delayed intubation, other delays, or a combination of these [5-7]. The ideal range for sleeve pressure on endotracheal cylinders is between 20 and 30 cm of H_2_O. High sleeve pressures can cause a variety of issues, such as a painful throat or hoarse voice, a narrowing of the trachea, putrefaction, or even a complete rupture of the trachea [8]. The tracheal mucosa is under such intense stress from the sleeve that it cannot receive adequate blood supply. If the sleeve pressure is inadequate, the patient may suction their stomach contents, which can cause end-stage pneumonitis and pneumonia. Several methods have been suggested to lessen the negative outcomes connected with mucosal irritation triggered by the endotracheal tube, including the administration of narcotics, extubation when the patient is under deep sedation, the use of fluticasone, and the infusion of intravenous lidocaine. Today's endotracheal tubes typically feature high-volume, low-pressure (HVLP) sleeves composed of polyvinyl chloride (PVC) or polyurethane (PU) [9]. When a high-volume, low-strain (HVLP) sleeve is inflated in the airway, the surplus material folds over itself to make channels, which can lead to a rupture [10-13]. Although it is undeniable that a smaller tracheal cylinder reduces the incidence of sore throat, perhaps due to reduced strain at the cylinder's mucosal contact, this method is not without its drawbacks. Post-extubation sore throat [6] has multiple possible causes but is typically related to laryngeal and pharyngeal damage. Post-extubation sore throat is also predicted by the female sex, younger patients, gynaecological operations, administration of succinylcholine, number of suctioning efforts, duration of intubation, number of manipulations needed to install the tube, and number of suctioning attempts [6-8,14].

The tapering curvature of the newly developed tracheal tube cuff made of polyvinyl chloride is an innovative method for minimizing fluid loss. The outside cuff diameter always matches the interior tracheal diameter because of the tapered cuff design. The area of the tracheal effect and the sleeve pressure needed to create a comfortable seal are both reduced with a tightly cinched sleeve endotracheal tube compared to a barrel-shaped sleeve endotracheal tube [15-19]. There is an additional benefit from a tapered cuff, according to the research, because the cuff's diameter gradually decreases to match the trachea's at one point [8]. By doing so, microaspiration is less likely to occur because longitudinal folds are less likely to form and a zone of full tracheal sealing is created [14]. This observational study compared the incidence of post-extubation sore throat, hoarseness of voice, and cough in surgical ICU patients intubated with a cylindrical- or tapered-shaped cuffed ETT.

## Materials and methods

This prospective, observational, longitudinal study was conducted by the Anesthesiology and General Surgery departments with approval from Dr. Balasaheb Vikhe Patil Rural Medical College Ethics Committee (approval no. RMC/UG-PG/2021/22, Dated 20/12/2021). Based on a previous study article by Chang et al. [12] on the incidence of sore throat we calculated the sample size using the formula developed by Lwanga and Lemeshow [16]. The sample size was calculated as 160 patients, with 80 patients in each group.

The formula used was, 

N= { Z √(2 P) (1-P) + Z 2 √(P1 (1- P1) - P2 (1- P2)2) } / (P1- P2 )2

Where P1: Probability of variable in sample-1 (value < 1.0)= 0.32, P2: Probability of variable in sample-2 (value < 1.0)= 0.54, P: Arithmetic average of P1 & P2= 0.43, AH: Arithmetic Hypothesis One-Sided = 2, 1-alpha: Set level of confidence ( usual values 0.95, 0.99)= 0.95, 1-beta: Set level of confidence ( usual values 0.8, 0.9)= 0.8, Z1: Z value associated with a set level of Alpha (one-sided): 1.959964, Z2: Z value associated with a set level of beta: 0.841621, N= minimum sample size obtained in each group was 79.

Patients older than 18 and below 57 years of either orientation who were intubated for more than 24 hours in the compassionate emergency unit were recalled for the concentrate after written informed consent was obtained. Patients who had been referred to our emergency clinic, who had a history of respiratory symptoms during the previous month, who required re-intubation within 24 hours of being extubated, and who had undergone an upper aviation route medical operation were not included in the evaluation. As per protocol, the ETT sleeve pressure was maintained at 25 cm of H_2_O by the diligent ICU nurses and doctors who monitored it regularly. Depending on the treating physician's preferences, a certain cylinder was employed. Patients in this review were divided into two groups by the names of Groups C and T: those who had been intubated with a sleeve endotracheal cylinder (Group C) and those who had had their sleeve endotracheal tube tightened (Group T). Details about intubation were noted, such as the number of attempts, the incubator's level of expertise, and the time it took to complete the procedure. Itching, coughing, and raspiness in the throat were among the side effects measured on a four-point scale [20]. At the 1, 12, and 24-hour marks following extubation, vital signs such as throat pain, dryness, and hacking were noted. The intubation duration, the intubating doctor's expertise, and the number of tries were among the non-mandatory results that were approximations. SPSS (Statistical Package for the Social Sciences) for Windows, version 20.0 (IBM Corp., Armonk, NY), was used for the empirical analysis. All quantitative data related to clinical markers were analyzed using the unpaired t-test (for contrasting two free samples). Based on anecdotal evidence, the Chi-square test was employed to compare and rank various gatherings. A value of 0.05 was selected as the threshold of statistical significance.

## Results

Our research population comprised 160 patients, with ages ranging from 18 to 57 (mean+SD: 45.53+19.947). Two groups (Group C and Group T) with 40 individuals each were created based on whether or not the endotracheal tube was used. Table 1 shows that there is no statistically significant difference in the distribution of ages and sexes between Group C and Group T (p>0.05).

**Table 1:** Table showing age and gender distribution

Age Distribution					
Groups	Mean	Standard Deviation	Minimum	Maximum	p value
Group C	45.53	19.94	18	87	0.08
Group T	40.98	12.83	19	73	
Gender Distribution					
Groups		Gender		Total	p value
		Female	Male		
Group C		39 (48.8%)	41 (51.2%)	80 (100.0%)	0.11
Group T		29 (36.2%)	51 (63.8%)	80 (100.0%)	

When comparing the number of tries and the level of experience of the residents who performed the intubations, there was no statistically significant difference between the groups (p>0.05) (Table 2).

**Table 2:** Number of intubation attempts and the experience of the person intubating the patients

		Number of intubating attempts					Total	p-value
		1st		2nd	3rd			
Groups	Group C	70 (87.5%)		9 (11.25%)	1 (1%)		80 (100.0%)	0.56
	Group T	74 (92.5%)		6 (7.5%)	0 (0%)		80 (100.0%)	
Experience of the person intubating								
			Group C			Group T		p-value
Junior Resident 1 year			28 (35%)			30 (37.5)		0.58
Junior Resident 2 year			40 (50%)			42 (52.5%)		
Junior Resident 3 year			12 (15%)			2 (2.5%)		

The time interval for post-extubation for hacking cough is shown in Table 3.

**Table 3:** Time interval from post-extubation for cough

Time	Group	0	1	2	3	p value
1^st^ Hour	Group C	2 (2.5%)	40 (50%)	30 (37.5%)	8 (10%)	0.37
	Group T	4 (5%)	50 (62.5%)	24 (30%)	2 (2.5%)	
12^th^ Hour	Group C	9 (11.25%)	47 (58.7%)	22 (27.5%)	2 (2.5%)	0.001
	Group T	27 (33.75%)	38 (47.5%)	14 (17.5%)	1 (1.25%)	
24^th^ Hour	Group C	38 (47.5%)	32 (40%)	10 (12.5%)	0 (0%)	0.43
	Group T	39 (48.75%)	37 (46.25%)	4 (5%)	0 (0%)	

**Figure 1:**
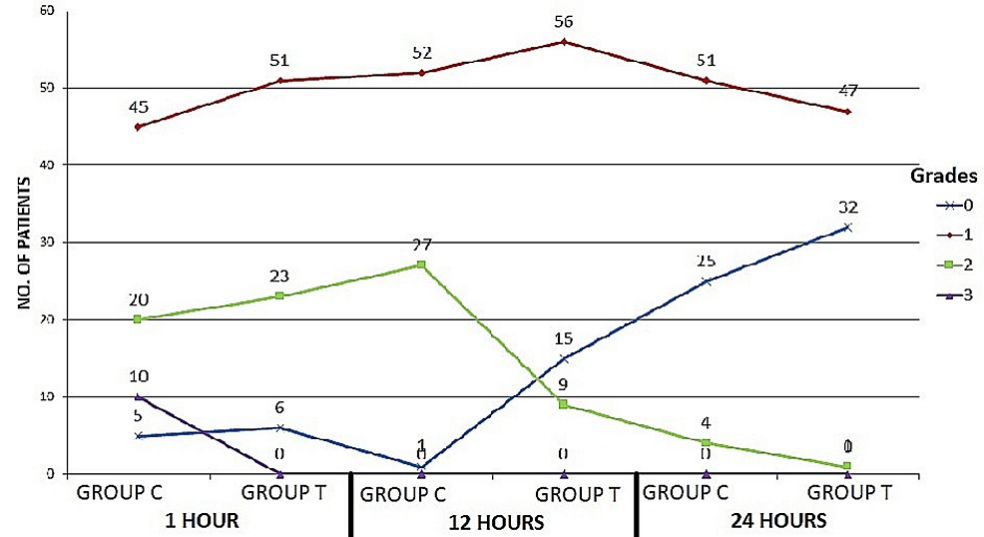
Grades of sore throat observed in Group C and T with respect to the time interval

Group T had lower rates of sore throat at 1, 12, and 24 hours after extubation compared to Group C ( Figure 1).

**Figure 2:**
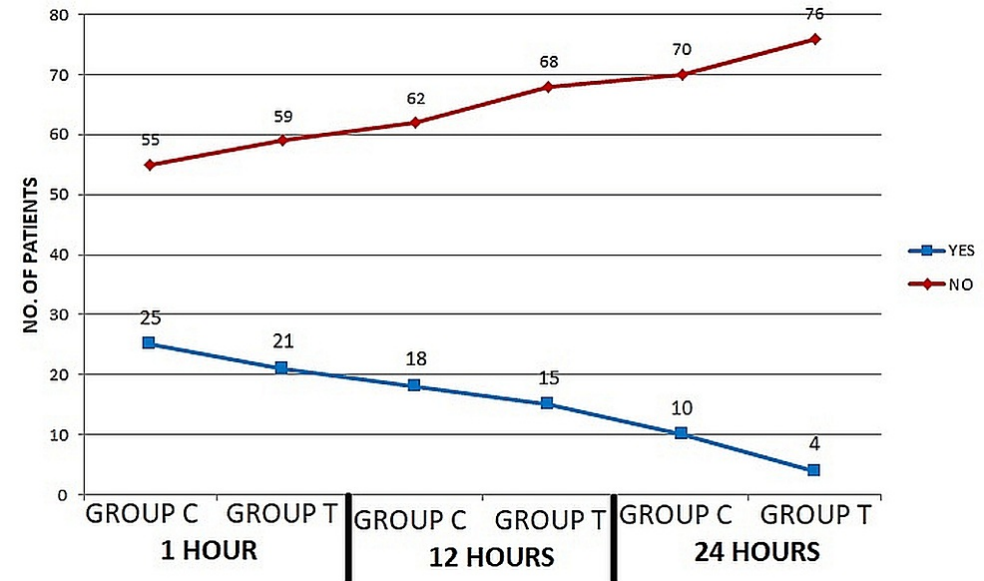
Time interval from post-extubation for hoarseness of voice

Hoarseness of voice after extubation decreased in frequency in both Group C and Group T with time (Figure 2).

**Figure 3:**
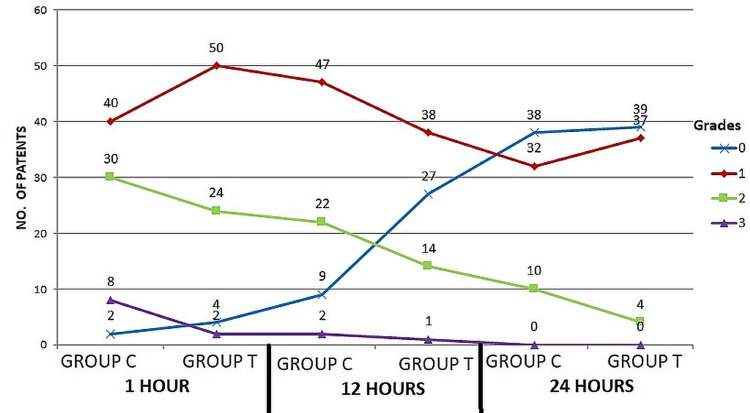
Time interval from post-extubation for cough

There is a declining trend in the incidence of cough post-extubation in Group T as compared to an initial increase in the trend for cough post-extubation with a gradual decline as time progressed in Group C (Figure 3).

## Discussion

Intubation is one of the most commonly performed invasive techniques in critical care units. It may be either elective or emergency. Various types of endotracheal tubes (ETT) are available in different sizes and shapes and developed with specific purposes. Various techniques are practiced to prevent the post-extubation emergence phenomenon, like the application of local anesthetics to the cuff, inflation of the cuff using only local anesthetics or with adjuncts like dexamethasone, sodium bicarbonate, etc. [20-22]. The ETT cuff's primary function is to create a tight enough seal to avoid aspiration while still allowing adequate blood flow to the trachea's mucosa. It is crucial to keep the cuff pressure of an endotracheal tube between 20 and 30 cm of H2O in order to avoid cuff-related problems in intubated patients [23]. Post-extubation sore throat, thought to be caused by tracheal mucosal erosion, was shown to be more common in patients with higher cuff pressure. According to research by Lakhe and Sharma, similar leakages of oropharyngeal secretions occur at lower pressures, such as 10 cm of H2O [14]. On an hourly basis, the nurse in our study checked the cuff pressure of each patient's endotracheal tube using an analog pressure gauge, ensuring that it remained at 25 cm of H2O throughout the study. The purpose of this study was to evaluate the effectiveness of a tapered cuff ETT against a cylindrical cuff ETT in preventing the emergence phenomena in surgically critical care patients. A tapered cuff ETT is associated with fewer cases of post-extubation sore throat, hoarseness of voice, and cough than a cylindrical cuff ETT, which was the primary finding of our study.

The Chi-square test was used to statistically compare the two groups' rates of post-extubation sore throat at different times. Most patients who have had a tracheostomy will complain of a sore throat when they are extubated. Post-extubation sore throat may be caused by irritation and inflammation caused by the endotracheal tube (ETT) remaining in the trachea after extubation. More cases of sore throat have been linked to tracheal intubation than to using a laryngeal mask airway or a facemask [16,17]. Sore throat symptoms were less common in Group T than in Group C at the 1-, 12-, and 24-hour post-extubation time points. The identical study found that in Group T, the incidence of sore throat decreased by six hours [12]. It's possible that our findings differ because we focused on critically ill surgical patients who were on prolonged intubation for far longer than the patients in the other trial.

The number of intubation attempts did not seem to have any bearing on the occurrence of throat difficulties in our study, which is consistent with the fact that we attempted many times for some patients in both Groups [13]. After 12 hours, the intensity of sore throats in the Gathering T group was significantly different from that in the Gathering C group (p<0.001). According to the most recent data by Chang et al., six hours after extubation, the incidence of sore throat was decreased in Gathering T compared to Gathering C (P = 0.006) but was not detected at the initial and 24-hour post-extubation evaluations. Five-sixths of patients intubated with a tracheal cylinder that had a tube-shaped sleeve had painful throat symptoms after 24 hours of extubation, according to the results of a study published in [12,16]. When the laryngeal branch of the external laryngeal nerve or the laryngeal branch of the internal laryngeal nerve is irritated by the endotracheal tube, paralysis of the cricothyroid muscle or the vocal folds occurs, respectively, leading to a scratchy voice. At 12 hours, the rate of recurrence of dryness after extubation was much lower in Gathering T (21) than in either Gathering C (26.25%) or Gathering C (31.25%). In contrast to what Yamanaka et al. [22] found about the severity of hoarseness after tracheal intubation, we did not find any evidence that age or length of intubation played a role in the persistence of dryness after extubation. The tapered cuff endotracheal tube was associated with significantly less severe hacking cough following extubation than a barrel-shaped sleeve endotracheal tube. Although the difference was clinically insignificant in the first and 24th hours significance was large in the 12th hour (p = 0.001) (Table 3).

After being freed from an endotracheal tube, many people experience throat pain, a change in their voice, and an increased tendency to hack. Research by Watchman et al. found that putting lidocaine into the endotracheal tube sleeve didn't reduce the incidence or severity of post-extubation sore throat compared to using saline or air. Pressure from a span sleeve applied after endotracheal intubation can help soothe a sore throat [23]. The use of steroid-coated endotracheal tubes and the inhalation of steroid medication are two strategies for protecting the pharynx, larynx, and windpipe against injury. Intravenous or topical ketoprofen has been shown to alleviate the discomfort of a sensitive throat after a tracheotomy or laryngoscopy is performed under general anesthesia [18].

Study limitations

This study is an observational, single-center study; hence the advantages and benefits of the tapered ETT cuff over the cylindrical type need to be established through additional randomized, prospective, blinded investigations. Neither the primary nor secondary goals were considered while documenting the size of the endotracheal tube. Finally, we could have followed up on both groups and examined the long-term effects.

## Conclusions

The use of tapered-guard endotracheal tubes among surgically critical patients reduces the overall incidence of the post-extubation emergence phenomenon when compared with the use of a conventional cylindrical-type endotracheal tube. Based on our study results, the side effects of prolonged intubation were significantly less during the first 24 hours of the extubation period except for the 12th hour compared to a barrel-shaped sleeve endotracheal tube. Thus in surgically critical patients, we recommend using a tapered-shape cuffed endotracheal tube rather than a cylindrical type.
